# The Rho ADP-ribosylating C3 exoenzyme binds cells via an Arg–Gly–Asp motif

**DOI:** 10.1074/jbc.M117.798231

**Published:** 2017-09-07

**Authors:** Astrid Rohrbeck, Markus Höltje, Andrej Adolf, Elisabeth Oms, Sandra Hagemann, Gudrun Ahnert-Hilger, Ingo Just

**Affiliations:** From the ‡ Institute of Toxicology, Hannover Medical School, Carl-Neuberg-Strasse 1, D-30625 Hannover and; the § Institute of Integrative Neuroanatomy, Charité-Universitätsmedizin, D-10115 Berlin, Germany

**Keywords:** ADP-ribosylation, bacterial toxin, ligand-binding protein, protein-protein interaction, vimentin, ADP-ribosyltransferase, C3 exoenzyme, RGD, binding, integrin

## Abstract

The Rho ADP-ribosylating C3 exoenzyme (C3bot) is a bacterial protein toxin devoid of a cell-binding or -translocation domain. Nevertheless, C3 can efficiently enter intact cells, including neurons, but the mechanism of C3 binding and uptake is not yet understood. Previously, we identified the intermediate filament vimentin as an extracellular membranous interaction partner of C3. However, uptake of C3 into cells still occurs (although reduced) in the absence of vimentin, indicating involvement of an additional host cell receptor. C3 harbors an Arg–Gly–Asp (RGD) motif, which is the major integrin-binding site, present in a variety of integrin ligands. To check whether the RGD motif of C3 is involved in binding to cells, we performed a competition assay with C3 and RGD peptide or with a monoclonal antibody binding to β1-integrin subunit and binding assays in different cell lines, primary neurons, and synaptosomes with C3-RGD mutants. Here, we report that preincubation of cells with the GRGDNP peptide strongly reduced C3 binding to cells. Moreover, mutation of the RGD motif reduced C3 binding to intact cells and also to recombinant vimentin. Anti-integrin antibodies also lowered the C3 binding to cells. Our results indicate that the RGD motif of C3 is at least one essential C3 motif for binding to host cells and that integrin is an additional receptor for C3 besides vimentin.

## Introduction

The family of bacterial C3^2^ exoenzymes comprises eight ADP-ribosyltransferases of different origin (1–7). *Clostridium botulinum* C3 transferase (C3) is the prototype of this family. It is a single chain protein of ∼25 kDa (8). C3 transfers an ADP-ribose moiety from NAD^+^ to the small GTPases RhoA, RhoB, and RhoC at asparagine 41, whereby RhoA is the preferred substrate (9). C3 structurally lacks a translocation and binding domain and also the crystal structure of C3 does not give any hints how binding to cells and uptake is mediated (10, 11). It has been postulated that C3 exoenzymes are nonspecifically taken up by target cells, due to a high concentration of C3 and extended incubation time (12, 13). Fusion of C3 to various types of transport peptides was also used to circumvent the lack of the canonical uptake domain of bacterial protein toxins (14–16). However, we and others have shown that C3 from *C. botulinum* and *Clostridium limosum* efficiently enter different cells (neurons, astrocytes, neutrophils, and macrophages) at nanomolar concentrations and within short time periods (17–21). Recently, we demonstrated that C3 entered Chinese hamster ovary (CHO) cells within 10 min at a C3 concentration of 100 nm (22). Additionally, vimentin was identified as cell surface binding partner of C3 (23).

RGD (Arg–Gly–Asp) is the major integrin binding motif and the minimal peptide region known to interact with subsets of integrins. The integrin family is composed of 18 α and 8 β subunits that form up to 24 different heterodimers (24). These integrin receptors form N-terminal extracellular domains that bind ligands to mediate extracellular signals into the cell (25). Various ligands have been reported to use the RGD motif for cell entry, for instance: collagen (26), fibronectin (27), osteopontin (28), and TAT protein of HIV-1 (29). Integrins are also known to serve as receptors for pathogens like *Yersinia pseudotuberculosis* invasin (30, 31), Herpes simplex virus type 1 glycoprotein H (32), Epstein-Barr virus (33), and human cytomegalovirus (34). Integrins are anchored by a transmembrane domain and interact with diverse cytosolic proteins such as talin by a short cytoplasmic tail (35, 36) and with filamin (37–39). Compelling evidence suggests that integrins also interact with vimentin (40–44). β3 integrin is associated with vimentin thereby recruiting vimentin to the cell surface (45). Vimentin is an intermediate filament mediating cell adhesion, migration (46–48), wound healing (49), and cellular signaling (50). Recent studies suggest that surface vimentin plays a role in uptake of several pathogens (51–56). The exact molecular mechanism how vimentin reaches the extracellular site of the plasma membrane remained unclear. Additional to integrin, vimentin can associate with numerous other proteins such as actin (57), tubulin (58, 59), filamin (60), soluble CD44 (61), and insulin-like growth factor 1 receptor (IGF1R) (62).

We previously identified a role for vimentin in binding and uptake of C3 (21). Disruption of the vimentin network through acrylamide or depletion of intracellular vimentin by siRNA clearly reduces C3 uptake but does not completely block the entry of C3 into cells (23). Recently, we showed in vimentin-knock-out neurons that vimentin is crucial for binding and uptake of C3 into neuronal cells (63). However, despite the complete lack of vimentin a weak signal of ADP-ribosylated RhoA/B was detected in vimentin-knock-out neurons. The extent of ADP-ribosylation was significantly reduced compared with the wild-type neurons but it was not completely inhibited. Additionally, application of extracellular vimentin to vimentin-knock-out neurons rescued the uptake of C3 and restored the growth-promoting effects of C3 (63). These findings suggested that C3 is able to bind to and enter cells via vimentin but another receptor appears to be involved.

In the present study we asked whether the RGD motif of C3 is relevant for its interaction with cell membranes. Indeed, we found that C3 binding to intact cells depends on the RGD motif. Moreover, results indicated that integrin and vimentin co-localized and interacted with each other. These findings suggest that integrin serves as a putative receptor for interacting with vimentin during C3 binding and uptake. Thus, both RGD motif and vimentin together with integrin are thought to mediate the uptake of C3.

## Results

### Reduced binding of C3 to vimentin-knock-out neurons

**Figure 1.**
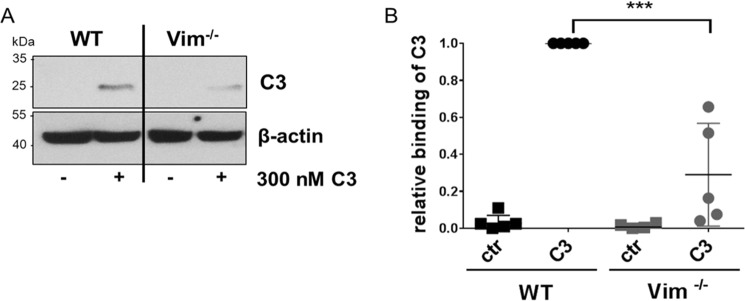
*A,* binding of C3 to intact primary vimentin-knock-out and wild-type neurons. Mixed hippocampal/neocortical neurons were exposed to 300 nm C3 for 1 h at 4 °C. Subsequently, cells were stringently washed three times, lysed, and submitted to Western blot analysis against C3 and β-actin. Western blots from representative experiments are shown (*n* = 5). *B,* diagram depicts densitometric evaluation of bound C3 and adjustment to the corresponding β-actin band. The signal intensity of bound C3 from C3-treated wild-type neurons was set as 1. Data represent the mean ± S.D. of five independent experiments. Statistical differences were determined by Student's *t* test (***, *p* ≤ 0.001).

Recently, we showed that vimentin is involved in binding and uptake of C3 into cells (23, 63). In this context we examined whether C3 binds to vimentin-knock-out neurons. Therefore, intact vimentin-knock-out and wild-type neurons were exposed to 300 nm C3 for 1 h at 4 °C followed by stringent washing, lysis, and submission to Western blot analysis against C3bot. Interestingly, C3 also bound to the vimentin-free (knock-out) neurons (Fig. 1*A*), however, to a significantly reduced amount (Fig. 1*B*). Thus, binding of C3 to cells is mediated by vimentin and an additional structure. Therefore, we performed a ClustalW alignment of C3 exoenzymes to identify a putative common sequence motif for a binding site. Sequence alignment revealed that C3bot, C3cer, C3lim, and C3larvin contain an Arg–Gly–Asp (RGD) integrin recognition motif in their N-terminal residues (aa 88–90) (supplemental Fig. S1, *A* and *B*).

### RGD peptide inhibits binding to and uptake of C3 into cells

Only a very limited quantity of neurons can be obtained from a single donor at one time and primary neurons cannot be expanded to large quantities, which are necessary for Western blot analysis. In addition, isolated primary neurons are often contaminated by astrocytes or glia cells. Thus, we used the immortalized murine hippocampal HT22 cell line as an alternative to primary neurons.

**Figure 2.**
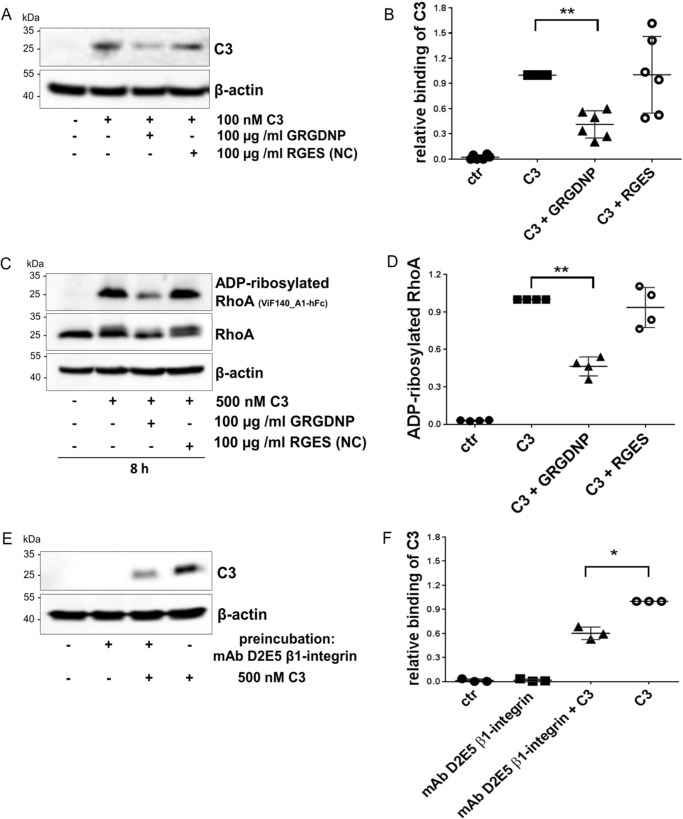
*A,* a competition assay with soluble RGD peptide revealed binding specificity of C3 to the RGD entity. HT22 cells were preincubated with 100 μg/ml of GRGDNP for 30 min at 4 °C followed by incubation with 100 nm C3 for 1 h at 4 °C. As negative control (*NC*) the RGES peptide was added. Subsequently, cells were stringently washed three times, lysed, and submitted to Western blot analysis against C3 and β-actin. Western blots from representative experiments are shown (*n* = 6). *B,* diagram depicts densitometric evaluation of bound C3 and adjustment to the corresponding β-actin band. The signal intensity of bound C3 from C3-treated cells was set as 1. Data represent the mean ± S.D. of six independent experiments. Statistical differences were determined by ANOVA and Dunnett's multiple *t* test (*, *p* ≤ 0.05; **, *p* ≤ 0.01). *C,* HT22 cells were preincubated with 100 μg/ml of GRGDNP for 30 min at 4 °C followed by incubation with 500 nm C3 for 8 h at 37 °C. Subsequently, cells were stringently washed three times, lysed, and submitted to Western blot analysis against RhoA, ADP-ribosylated RhoA, and β-actin. The signal intensity of ADP-ribosylated RhoA from C3-treated cells (without preincubation) was set as 1. Western blots from representative experiments are shown (*n* = 4). *D,* densitometric analysis of (*C*) ADP-ribosylated Rho antibody is shown. Data represent the mean ± S.D. of four independent experiments. Statistical differences were determined by ANOVA and Dunnett's multiple *t* test (*, *p* ≤ 0.05; **, *p* ≤ 0.01). *E,* HT22 cells were preincubated with 3 μl/ml of mAb D2E5 to the β1-integrin subunit for 30 min at 4 °C followed by incubation with 500 nm C3 for 1 h at 4 °C. Subsequently, cells were stringently washed three times, lysed, and submitted to Western blot analysis against C3 and β-actin. Western blots from representative experiments are shown (*n* = 3). *F,* diagram depicts densitometric evaluation of bound C3 and adjustment to the corresponding β-actin band. The signal intensity of ADP-ribosylated RhoA from C3-treated cells (without preincubation with the antibody) was set as 1. Data represent the mean ± S.D. of three independent experiments. Statistical differences were determined by ANOVA and Dunnett's multiple *t* test (*, *p* ≤ 0.05).

To investigate whether the RGD motif of C3bot is involved in binding and uptake into HT22 cells, a RGD competition assay was performed. Short synthetic peptides containing the RGD motif were used to inhibit binding to integrin (Fig. 2*A*). Binding of C3 to intact HT22 cells were competed by the G**RGD**NP peptide. A concentration of 100 μg/ml of G**RGD**NP peptide resulted in an approximate 50% displacement of C3 from the HT22 cells (Fig. 2*B*). Furthermore, preincubation of cells with G**RGD**NP peptide strongly inhibited internalization of C3 as evidenced by a diminished RhoA shift and decreased amount of ADP-ribosylated RhoA (Fig. 2, *C* and *D*). To confirm this finding, cells were pretreated with mAb D2E5, a monoclonal antibody that binds to the β1-integrin subunit followed by incubation with C3 for 1 h at 4 °C. Antibody treatment strongly reduced binding of C3 to intact cells (Fig. 2, *E* and *F*) and supported the findings of the competition assay with the RGD peptide.

### Role of RGD motif in C3 binding to cells

The RGD sequence of C3 was mutated to RGG (D90G) or RGN (D90N) as well as RID (G89I). Because the arginine (Arg-88) residue of the RGD motif is important for the C3–NAD interaction (10, 64) this amino acid was not changed. The Asp-90 of the RGD motif forms a hydrogen bond to Arg-88 and therefore stabilizes the C3–NAD complex (10). Therefore, we performed two different Asp-mutant forms and converted the negatively charged Asp-90 to an uncharged Asn-90 or uncharged neutral Gly-90. No function of Gly-89 within the RGD motif has been reported.

**Figure 3.**
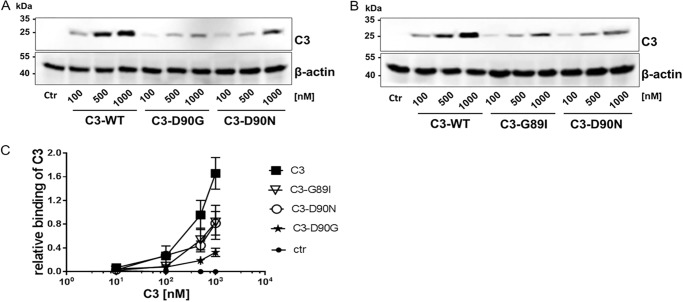
*A,* HT22 cells were exposed to increasing concentrations of C3 and C3-RGD mutants (C3-D90G and C3-D90N) and C3-G89I (*B*) for 1 h at 4 °C. Subsequently, cells were stringently washed three times, lysed, and submitted to Western blot analysis against C3 and β-actin. C3 and C3-D90N were analyzed in duplicates (*A* and *B*). *C,* diagram depicting densitometric evaluation of bound C3 and adjustment to the corresponding β-actin band (*n* = 5). Results represent the arithmetic mean ± S.D. of five independent experiments. Statistical differences between C3 and C3-RGD mutant-treated cells were determined by ANOVA and Dunnett's multiple *t* test. Results were statistically significant relative to C3 at a concentration of 500 and 1000 nm value, *p* ≤ 0.05.

Binding assays in three different cell types were performed to evaluate the influence of the RGD, RGN, or RID mutation in C3 on cell binding. Notably, recognition of C3, C3-D90G, C3-D90N, and C3-G89I by α-C3bot was comparable (supplemental Fig. S2). Therefore, intact cells were exposed to increasing concentrations of C3 or C3-RGD mutants (10, 100, 500, and 1000 nm) for 1 h at 4 °C, followed by stringent washing, lysis, and submission to Western blot analysis against α-C3bot. Fig. 3, *A* and *B*, shows that C3 and the C3-RGD mutants bound to intact HT22 cells in a concentration-dependent manner. A significantly reduced binding of C3-RGD mutants compared with C3 wild-type was observed. Interestingly, the C3-D90G mutant bound less than the C3-G89I and C3-D90N mutants (Fig. 3*C*). The binding data were confirmed in macrophage-like cell line J774A.1 (supplemental Fig. S3, *A–C*) and also in hamster ovary CHO cells (supplemental Fig. S3, *D–F*). In both cell lines a strongly reduced binding of C3-RGD mutants compared with C3 was detected. Noteworthy, in hamster CHO cells the C3-G89I mutant bound weaker than the C3-D90N mutant. These findings indicate that the RGD motif of C3 is important for binding to intact cells.

### Role of RGD motif in C3 binding to recombinant vimentin

**Figure 4.**
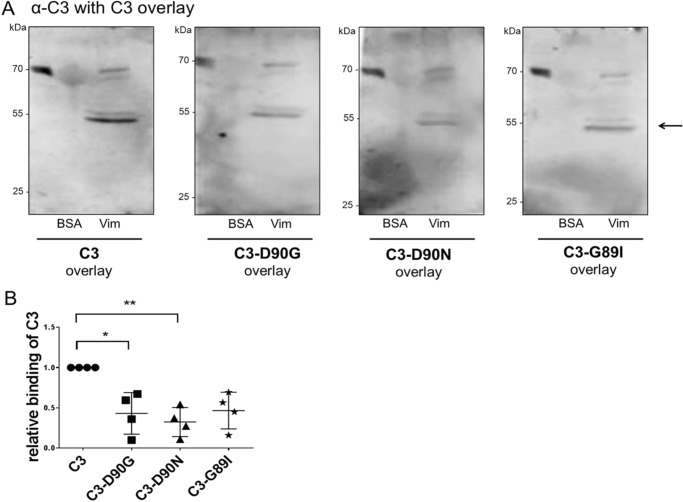
*A,* C3 overlay (binding of C3 to vimentin). Recombinant vimentin (35 μg) was purified as described under ”Experimental procedures“ followed by separation through SDS-PAGE and transfer onto nitrocellulose. Nitrocellulose was incubated with 10 μg/ml of C3 or C3-RGD mutants (as indicated) for 1 h at 4 °C. After washing, bound C3 was detected by anti-C3. BSA was used as negative control. *B,* diagram depicts densitometric evaluation of bound C3 and adjustment to the 70-kDa reference band of the protein ladder (*n* = 4). The signal intensity of bound C3-WT was set as 1. Data represent the mean ± S.D. of four independent experiments. Statistical differences were determined by ANOVA and Dunnett's multiple *t* test (*, *p* ≤ 0.05; **, *p* ≤ 0.01).

**Figure 5.**
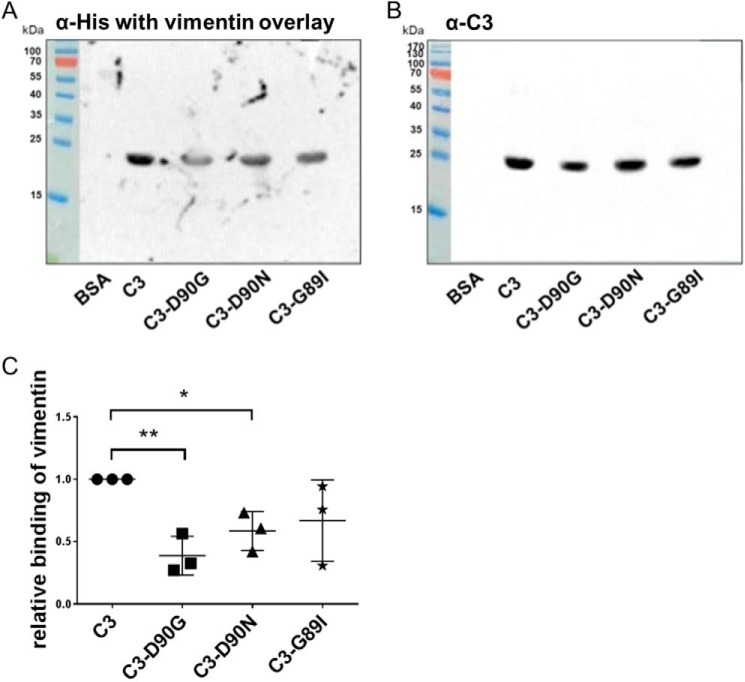
*A,* binding of vimentin to C3 or C3-RGD mutants (as indicated). Purified C3 (4 μg) or C3-RGD mutants (4 μg) were separated through SDS-PAGE and transfered onto nitrocellulose. Nitrocellulose was incubated with 10 μg of His-tagged vimentin for 60 min at 4 °C. After washing, bound vimentin was detected by penta-His polyclonal antibody. BSA was used as negative control. *B,* Western blot of loading C3 samples is shown. *C,* diagram depicts densitometric evaluation of bound vimentin and adjustment to the corresponding band in the α-C3 Western blot (*n* = 3). The signal intensity of bound His-tagged C3-WT was set as 1. Data represent the mean ± S.D. of three independent experiments. Statistical differences were determined by ANOVA and Dunnett's multiple *t* test (*, *p* ≤ 0.05; **, *p* ≤ 0.01).

In previous studies we showed that vimentin mediates binding and uptake of C3 into cells. To study whether the RGD motif of C3 is involved in binding to vimentin, recombinant vimentin was separated by SDS-PAGE followed by electroblotting onto nitrocellulose. The nitrocellulose was then overlaid with C3 or the C3-RGD mutants (C3-D90G, C3-D90N, or C3-G89I) followed by washing and detecting C3 by α-C3bot. Bound C3 was detected at a molecular mass of about 55 kDa (Fig. 4*A*, *arrow*). Binding of C3 seemed to be specific, because no signal was detected with BSA, which was loaded as negative control. Densitometric evaluation of bound C3 showed that wild-type C3 bound most strongly to vimentin, followed by C3-G89I, which bound about 45% less than C3. The substitution of asparagine at amino acid position 90 also caused a reduced binding capacity of C3-D90G and C3-D90N to vimentin (Fig. 4*B*). To rule out that any mutation in C3 *per se* altered the binding of C3 to vimentin, we performed the C3 overlay experiment with the enzyme-deficient C3-E174Q (supplemental Fig. S4*A*). In this assay the HT22 cell lysate served as positive control and showed that binding of C3-E174Q to the HT22 cell lysate and recombinant vimentin was similar to binding of C3 (supplemental Fig. S4*B*). To confirm this finding a reverse experiment was set up. C3 and C3-D90G, C3-D90N, and C3-G89I were separated by SDS-PAGE, blotted onto nitrocellulose membrane, and overlaid with His-tagged vimentin. After intensive washing the vimentin protein was visualized with an anti-His antibody (Fig. 5*A*). The loaded C3 were visualized using the C3bot antibody (Fig. 5*B*). Densitometric evaluation of bound His-tagged vimentin confirmed interaction between vimentin and C3, as well as C3-G89I and C3-G90N (Fig. 5*C*), however, with a reduced binding of vimentin to the C3-RGD mutants. Taken together, these results strongly indicate that the RGD motif is involved in binding of C3 to vimentin.

To study if the amino acid substitutions within the RGD motif impair the ability of C3 to ADP-ribosylate RhoA we performed an *in vitro* sequential [^32^P]ADP-ribosylation assay with recombinant RhoA. The enzyme activity of all three C3-RGD mutants was significantly reduced (data not shown), which hampers the statement about the uptake of the C3-RGD mutants.

### Synaptosomes

**Figure 6.**
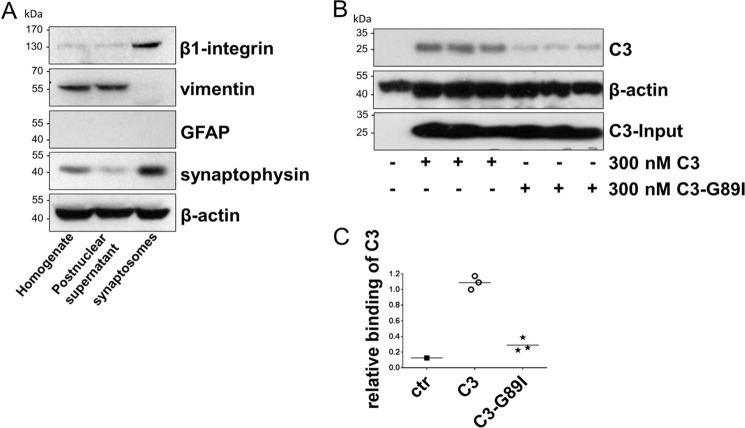
*A,* total protein (20 μg) from mouse brain homogenates, postnuclear supernatant, and synaptosome suspension were prepared and submitted to Western blot analysis against the synaptic protein synaptophysin, β1-integrin, the intermediate filaments vimentin and glial fibrillary acidic protein (*GFAP*). β-Actin served as a loading control. *B,* synaptosomes were incubated with 300 nm C3 or C3-G89I for 1 h at 4 °C. Subsequently, synaptosomes were stringently washed, lysed, and submitted to Western blot analysis against C3 and β-actin. *C,* binding of C3 is shown as means of three biological triplicates (*n* = 1). Bound C3 was densitometrically evaluated and adjusted to the corresponding β-actin and C3-input band.

To confirm that β1-integrin is involved in binding of C3 to cells, we conducted further experiments using synaptosomes prepared from postnatal mouse whole brains. Western blotting detected the presynaptic marker protein synaptophysin enriched in synaptosomes compared with whole brain homogenate and postnuclear supernatant. Also, β1-integrin was detected in synaptosomes at significantly higher levels. The intermediate filament protein vimentin, which is expressed in adult brains mainly in glial cells but not in neurons, was detected in the homogenate and postnuclear supernatant but not in synaptosomes (Fig. 6*A*). Both the lack of vimentin and the absence of glial fibrillary acidic protein hardly detectable in young postnatal (P2) brains indicated a successful depletion of the synaptosomal fraction from glial constituents. Therefore, synaptosomes represent a suitable purified neuronal model to evaluate the binding of C3 to integrin. Despite the lack of vimentin, C3 bound to synaptosomes and confirmed the observed binding of C3 to vimentin-knock-out neurons (see Fig. 1). Substitution of Gly of the RGD motif resulted in strong reduction of C3-G89I binding to synaptosomes (Fig. 6, *B* and *C*). This finding supports the notion that the RGD motif is involved in binding of C3 to cells including neurons. Notably, a direct comparison of the influence of vimentin on C3-binding is not possible, because synaptosomes do not contain vimentin.

### Reduced binding of His-C3stau2 to cells compared with His-C3bot

**Figure 7.**
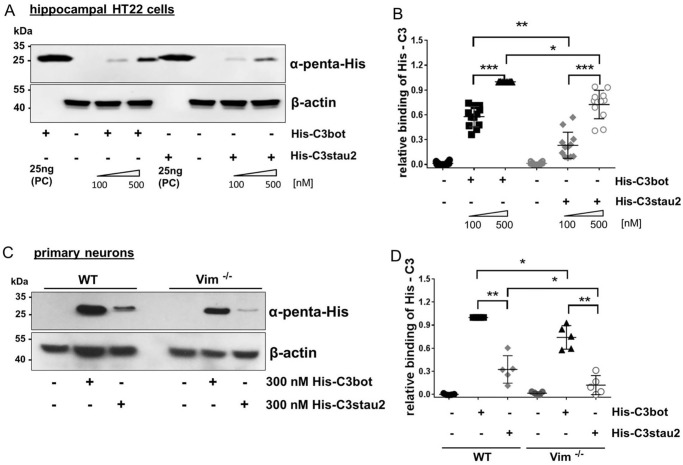
*A,* binding of His-C3bot and His-C3stau2 to intact hippocampal HT22 cells. Cells were exposed to 100 or 500 nm His-C3bot or His-C3stau2 for 1 h at 4 °C. Subsequently, cells were stringently washed three times, lysed, and submitted to Western blot analysis against penta-His and β-actin. Western blots from representative experiments are shown (*n* = 5). *PC,* positive control, 25 ng of His-C3bot or His-C3stau2 in 5× Laemmli buffer. *B,* diagram depicts densitometric evaluation of bound His-tagged C3 and adjustment to the corresponding β-actin band. The signal intensity of bound His-C3 from 500 nm His-C3bot-treated HT22 cells was set as 1. Data represent the mean ± S.D. of five independent experiments with biological duplicates. Statistical differences were determined by ANOVA and Dunnett's multiple *t* test (*, *p* ≤ 0.05; **, *p* ≤ 0.01; ***, *p* ≤ 0.001). *C,* binding of His-tagged C3 to intact primary vimentin-knock-out and wild-type neurons. Mixed hippocampal/neocortical neurons were exposed to 300 nm His-C3bot or His-C3stau2 for 1 h at 4 °C. Subsequently, primary neurons were stringently washed, lysed, and submitted to Western blot analysis against penta-His and β-actin. *D,* diagram depicts densitometric evaluation of bound His-tagged C3 and adjustment to the corresponding β-actin band. The signal intensity of bound His-C3 from 300 nm His-C3bot-treated wild-type neurons was set as 1. Data represent the mean ± S.D. of five independent experiments. Statistical differences were determined by ANOVA and Dunnett's multiple *t* test (*, *p* ≤ 0.05; **, *p* ≤ 0.01).

Sequence alignment revealed that all three isoforms of C3stau harbor instead of the RGD motif a RGD-like motif, Arg–Leu–Asp (RLD aa 70–72) sequence, which is recognized by αvβ3 and α_M_β2 integrins (65, 66). Additionally, C3stau contains a LLNLD (aa 89–93) sequence (supplemental Fig. S1, *A* and *C*), which is consistent with the integrin α subunit recognition consensus (67) and represents a clathrin box motif (68). To investigate the C3 cell interaction further, a His-tagged form of the C3bot and C3stau2 exoenzymes was used (His-tagged C3 proteins were used due the lack of a suitable antibody recognizing both C3bot and C3stau2 at comparable affinities). To investigate binding of the C3 exoenzymes to HT22 cells, identical amounts of His-C3bot or His-C3stau2 were added to cells for 1 h at 4 °C. Significant differences between His-C3bot and His-C3stau2 binding to cells were observed. His-C3stau2 binding to HT22 cells was reduced compared with binding of His-C3bot (Fig. 7*A*). Binding of His-C3stau2 to HT22 cells was ∼60% of the binding of His-C3bot (Fig. 7*B*). In addition, a reduced binding of His-C3stau2 in primary cells was detected (Fig. 7*C*). Moreover, vimentin mediates binding of both His-C3bot and His-C3stau2 because the amount of bound His-C3 to vimentin-knock-out neurons was significantly reduced compared with wild-type neurons. Finally, a reduced binding of His-C3stau2 compared with His-C3bot was detected in vimentin-knock-out neurons (Fig. 7*D*). These differences suggest that the altered binding site of C3stau2 might result in reduced binding affinity.

## Discussion

Previous studies have revealed that vimentin is involved in binding and uptake of C3bot (23). However, findings indicate that alternative or additional binding partners besides vimentin may exist. First, although vimentin was successfully reduced by siRNA, binding and uptake of C3bot was not completely inhibited. Second, in C3 overlay-binding assays several positive spots were detected containing other proteins than vimentin. Third, as shown in Figs. 1, 6*B,* and 7*C* despite the lack of vimentin, C3 binds to vimentin-knock-out neurons and vimentin-free synaptosomes. To identify additional binding partners via binding consensus sequences or binding motifs in C3 we performed sequence alignment analyses of different C3 isoforms and found the RGD motif in all C3 exoenzymes with the exception of C3stau. The RGD motif is the cell attachment site of a large number of adhesive extracellular matrix, blood, and cell surface proteins, and known integrins (αvβ1, αvβ3_,_ αvβ5_,_ αvβ8, and α5β1) recognize this sequence. Studies have shown that many pathogens use different types of RGD-recognizing integrin receptors (αvβ1, αvβ3, αvβ6, and αvβ8) to bind to and enter the host cells (69–72). The RGD motif has also been found in bacterial protein toxins. For example, the α-toxin of *Staphylococcus aureus* interacts with β1-integrin of epithelial cells (73). The *Streptococcus pyogenes* pyrogenic exotoxin B binds to αvβ3 or α2bβ3 integrins on endothelial cells and platelets (74). The role and importance of the RGD motif in binding of C3 to cells has been investigated by competition-binding studies. The competitive inhibition experiments using the G**RGD**NP peptide and preincubation of cells with an antibody against β1-integrin remarkably decreased the binding of C3 to HT22 cells. This indicates that the RGD motif is crucial for effective interaction between C3 and HT22 cells. Additionally, mutations in the RGD motif of C3 (such as Asp-90 and Gly-89) led to reduced binding of C3 to different cell lines. But the replacement of this amino acid also leads to significant loss of enzymatic activity as this motif is also involved in binding to the cosubstrate NAD^+^.

Furthermore, compelling evidence suggests that integrins associate with vimentin. The αvβ3 and α2β1 integrins bind to vimentin through integrin-associated proteins (41). In primary microvascular endothelial cells fibrinogen-associated αvβ3 integrin was assembled in structures harboring vimentin filaments (42). In CHO cells Bhattacharya and co-workers (45) demonstrated a close association between β3 integrin and vimentin at the basal surface of the cell. Moreover, they showed that integrin regulates interaction of vimentin with the cell surface. Recently, it was also shown that vimentin filaments underneath the plasma membrane directly interact with integrin tails and cause integrin clustering and enhanced integrin-mediated cell adhesion (44). Integrins are transmembrane cell adhesion receptors; therefore the interaction between vimentin and integrin takes place near the plasma membrane. From these data we suggest that vimentin from the plasma membrane region interacts with integrin. Interestingly, vimentin contains an Arg–Leu–Asp (RLD) motif within the rod1b domain (aa 217–219). RLD is a binding motif for integrins αvβ3 and α_M_β2 (65, 69, 75). Based on this finding an extracellular interaction between vimentin and integrin appears feasible. In a previous study the rod domain of vimentin was identified as a binding partner of C3 (23). Vimentin at the cell surface has been reported in several studies (23, 76–80). Vimentin is involved in binding and uptake of several pathogens (51–54, 81, 82). Recently, a further study has shown that surface vimentin is a co-receptor for SARS-coronavirus (56). Moreover, findings indicated that vimentin is one of the interacting partners of the ACE2 receptor (the identified functional receptor for SARS-coronavirus, (83)) and directly binds to the SARS-coronavirus spike protein. Vimentin also mediates the entry of SARS-coronavirus (56). In this context, it is known that in addition to integrins, other proteins or receptors served as co-receptor and were involved in cell entry of pathogens. For example, both integrins (α3β1 and α9β1) and ephrin receptor tyrosin kinase A2 serve as receptors for Kaposi's sarcoma-associated herpesvirus (84–86). Like Kaposi's sarcoma-associated herpesvirus, adenovirus type 2 uses coxsackievirus and adenovirus receptor (CAR) and αvβ3/αvβ5 integrins for productive entry (87, 88). Furthermore, reovirus binds first to its cell attachment factor sialic acid (89) and then to its co-receptor β1 integrin (90). This interaction drives the recruitment and clustering of the junctional adhesion molecule A and caused internalization of the virus–receptor complex (91, 92). However, both vimentin and integrin have been shown to act as direct receptor and co-receptor and both are involved in endocytosis. Therefore, it is conceivable that vimentin and integrin also act together. This is supported by the findings of Yang and co-worker (93), they described that the rod domain of superficial vimentin is involved in binding and uptake of dengue virus. Previously it was shown that β3 integrin was required for dengue virus serotype 2 infection (94). Therefore it was postulated that dengue virus uses more than one receptor to infect cells and that perhaps β3 integrin and superficial vimentin cooperate in mediating dengue virus infection (93).

The involvement of the RGD motif in binding to cells is clear. As mutations within the RGD motif of C3 cause loss of enzyme activity, no study of the role of the RGD motif in the uptake of C3 can be performed. In conclusion, the RGD motif in C3 is crucially involved in its binding to cells, as even conservative changes to RGG, RGN, or RID significantly reduced the binding capacity of C3. The RGD motif mediates direct binding of C3, to recombinant vimentin as well as to integrin (synaptosomes, Vim^−/−^ neurons). Moreover, His-C3bot binding to cells was significantly enhanced compared with His-C3stau2, which lacks the RGD motif. C3bot harbors only one RGD motif implicating binding to either vimentin or integrin. However, a complex between vimentin and β1 integrin may enhance the C3bot binding. How the interaction of vimentin with integrin is regulated by C3 is still unclear. But all three players must cooperate as addition of vimentin to vimentin-knock-out neurons (harboring integrin) enhances strong binding and uptake of C3bot (63).

## Experimental procedures

### Cell culture

Murine hippocampal HT22 cells, which were a generous gift from Prof. Dr. Carsten Culmsee (Institute for Pharmacology and Toxicology, Philipps University Marburg, Germany) (95), were cultured in Dulbecco's modified essential medium (Biochrom, + 10% FCS, 1% penicillin, 1% streptomycin, and 1 mm sodium pyruvate). J774A.1 mouse macrophages (purchased from American Type Culture Collection ATCC: TIB-67) were cultivated in RPMI 1640 medium (Biochrom; with 10% FCS, 1% penicillin, 1% streptomycin, and 1 mm sodium pyruvate). Cells were maintained at 37 °C and 5% CO_2_. Upon confluence, cells were passaged. Wild-type Chinese hamster ovary cells (CHO-K1, ATCC: CCL-61, which were a generous gift from Prof. Dr. Gerardy-Schahn (Institute for Cellular Chemistry, Hannover Medical School, Germany) were cultivated in Dulbecco's modified essential medium/Ham's F-12 medium (Biochrom; with 10% fetal bovine serum, 1% penicillin, 100 units/ml of streptomycin, and 1 mm sodium pyruvate). Cells were maintained at 37 °C and 5% CO_2_. Upon subconfluence, cells were passaged. Neurons were obtained from fetal NMRI (hippocampus culture) or 129 SVEV wild-type and 129 SVEV Vim^−/−^ knock-out mice (mixed hippocampal/neocortical culture) at embryonic day 16 (E16) as described before (63).

Synaptosomes were prepared at 4 °C from postnatal (P2) mouse whole brains in the presence of protease inhibitors according to a modified standard procedure as described (96). Briefly, after homogenization in 0.32 m sucrose (10 strokes at 900 rpm) and centrifugation at 1,300 × *g* for 10 min, the supernatants were centrifuged at 14,000 × *g* for 15 min. Crude synaptosomes (1–2 mg of protein) were dissolved in PBS and incubated with C3 or C3-G89I.

### RGD competition assay

For the RGD competition assays cultured HT22 cells were seeded onto 3.5-cm plates at a concentration of 300,000 cells/ml and grown for 24 h at 37 °C and 5% CO_2_. The medium was removed and cells were washed with PBS. HT22 cells were preincubated with 100 μg/ml of G**RGD**NP for 30 min at 4 °C followed by incubation with 100 nm C3 for 1 h at 4 °C. As negative control the RGES peptide was added. Cells were washed and scraped into Laemmli sample buffer. The obtained suspension was shaken at 37 °C for 10 min. Ultrasonic disruption was performed in a cycle of 10 × 5 s, 5 × 10% sonic energy using a sonotrode (Bandelin Electronic, Berlin, Germany). The lysate was then incubated at 95 °C for 10 min and submitted to SDS-PAGE and Western blot analysis against α-C3 and β-actin.

### Expression and purification of recombinant C3 protein

C3 wild-type and *C. botulinum*-derived mutant C3-E174Q (carrying a point mutation from glutamate to glutamine at amino acid 174) were expressed as recombinant GST fusion proteins in *Escherichia coli* TG1 harboring the respective DNA fragment (gene of *C. botulinum* C3, accession number X59039) in plasmid pGEX-2T (GE Healthcare Europe GmbH, Freiburg, Germany) as described previously. Briefly, pGEX-2T plasmids encoding wild-type or enzyme-deficient C3bot were transformed into *E. coli* TG1 cells. Starter cultures grown in Luria-Bertani broth with ampicillin at 37 °C overnight were diluted into fresh media. Cells were grown for 3 h (*A*_600_ = 0.7) at 37 °C, isopropyl 1-thio-β-d-galactopyranoside was added to a final concentration of 200 μm to induce the C3bot expression for another 3 h. Cells were harvested by centrifugation, resuspendend in lysis buffer (20 mm Tris-HCl, pH 7.4, 10 mm NaCl, 5 mm MgCl_2,_ 5 mm DTT, and 1 mm PMSF), and lysed ultrasonically (3 × 20 s, 90% cycle, 20% power) on ice. The lysate was centrifuged for 30 min at 15,000 × *g*. The supernatant was incubated with glutathione-Sepharose beads for 5 h at 4 °C to bind the GST fusion protein. Beads were washed with buffer containing 50 mm Tris-HCl (pH 8.0), 10 mm glutathione, 100 mm NaCl and PMSF. The GST-fused C3bot protein was cleaved from the beads with thrombin (Sigma, Berlin, Germany) in a buffer containing 50 mm Tris-HCl (pH 8.0), 50 mm NaCl, and 2.5 mm CaCl_2_. Purified proteins were then concentrated to 1 ml with a Centricon microconcentrator (Amicon, Danvers, MA) with a 30,000 molecular weight cutoff. Thrombin was removed from purified C3bot by use of *p-*aminobenzamidine beads (Sigma). Buffer exchange was performed by use of PD 10 column (GE Healthcare, Europe GmbH, Freiburg, Germany) and purified C3bot was eluted in 20 mm HEPES (pH 7.5). Eluted proteins were analyzed by 15% SDS-PAGE and stained with Coomassie Blue. ADP-ribosyltransferase activity was measured by an *in vitro* ADP-ribosylation assay.

### Generation of C3-RGD mutants by site-directed mutagenesis

Individual substitution of asparagine or glycine residues within the RGD motif of C3bot were introduced using QuikChange II XL site-directed mutagenesis kit, according to the manufacturer's instructions (Agilent, Santa Clara, CA). The primers used were as follows: forward, D90G_For (5′-ATGTTATTTAGAGGCGGCGACCCTGCTTATTTAGG-3′), and reverse, D90G_Rev (5′-CCTAAATAAGCAGGGTCGCCGCCTCTAAATAACAT-3′), D90N_For (5′-ATGTTATTTAGAGGCAACGACCCTGCTTATTTAGG-3′), and reverse, D90N_Rev (5′-CCTAAATAAGCAGGGTCGTTGCCTCTAAATAACAT-3′), and G89I_For (5′-ATGTTATTTAGAATCGACGACCCTGCTTATTTAGG-3′), and reverse, G89I_Rev (5′-CCTAAATAAGCAGGGTCGTCGATTCTAAATAACAT-3′). Correct nucleotide sequences were confirmed by sequencing.

### Expression and purification of His-tagged recombinant C3 proteins

C3bot (gene of *C. botulinum* C3, accession number X59039) and C3stau2 (gene of *Staphylococcus aureus* C3, accession number AJ277173) cDNA constructs were successfully cloned into pQE-30 vector (Qiagen), with the N-terminal His tag. The recombinant constructs were transformed into *E. coli* TG1 and grown in 1-liter volumes of LB medium. The cells were grown to an *A*_600_ of 0.6 and induced with 200 μm isopropyl 1-thio-β-d-galactopyranoside for 3 h. Cells were collected by centrifugation (5,000 rpm for 20 min) and kept at −20 °C overnight. The frozen cells were later thawed, resuspended with 50 ml of lysis buffer (50 mm NaH_2_PO_4_, 300 mm NaCl, and 10 mm imidazole), disrupted using a French press, and centrifuged at 15,000 rpm for 20 min. The resultant supernatant was incubated for 30 min on ice with DNase (10 μg/ml) and loaded onto a Talon metal affinity column (BD Biosciences Clontech, BD Biosciences). Bound His-tagged C3 protein was eluted with elution buffer (50 mm NaH_2_PO_4_, 300 mm NaCl, and 250 mm imidazole, adjust to pH 8.0). Buffer exchange was performed by use of PD 10 column (GE Healthcare, Europe GmbH) and purified His-tagged C3 was eluted in 20 mm HEPES (pH 7.5). Eluted proteins were analyzed by 15% SDS-PAGE and stained with Coomassie Blue. ADP-ribosyltransferase activity was measured by an *in vitro* ADP-ribosylation assay. Protein concentrations were determined using the NanoDrop ND-1000 spectrophotometer.

### C3 binding assay

For the binding assays cultured cells were seeded onto 3.5-cm plates at a concentration of 300,000 cells/ml and grown for 24 h at 37 °C and 5% CO_2_. The medium was removed and cells were washed with PBS. 300,000 cells/ml were exposed to 10, 100, 500, or 1000 nm C3 or C3-RGD mutants for 1 h at 4 °C. Subsequently, cells were stringently washed three times with PBS. Cells were scraped into Laemmli sample buffer.

### Western blot analysis

For Western blot analysis the following primary antibodies were used: RhoA was identified using a mouse monoclonal IgG from Santa Cruz Biotechnologies (catalog number sc-418). Identification of C3 was achieved by a rabbit polyclonal antibody (affinity purified), which was raised against the full-length toxin C3bot (accession number CAA41767). ADP-ribosylated RhoA was detected by a specific antibody against ADP-ribosylated RhoA (ViF140_A1-hFc antibody was kindly provided by Viola Fühner and Michael Hust, Technische Universität Braunschweig, Germany (22)). β-Actin was identified using mouse monoclonal anti-actin antibody (catalog number A5441, Sigma). Vimentin was identified using rabbit monoclonal anti-vimentin antibody (catalog number ab92547, Abcam, Cambridge, UK). α-Penta-His (catalog number 34660, Qiagen, Hilden, Germany) antibody was used to detect His-tagged vimentin. Western blot analyses were performed as described previously (23).

### C3 overlay assay

35 μg of recombinant vimentin were separated by 15% SDS-PAGE and transferred to nitrocellulose membranes. After Western blot the transfer efficiency was checked with Ponceau S staining. After incubation with blocking buffer (5% powdered milk, in Tris-buffered saline with Tween (TBST)), membranes were probed with 10 μg/ml of purified C3 or C3-RGD mutants in blocking buffer 1 h at 4 °C. After extensive washing with TBST, membranes were incubated with C3 antibody, followed by HRP-conjugated secondary antibody (catalog number 611-1302, Rockland Immunochemicals Inc., Pottstown, PA), and detected by ECL. As a negative control, 2 μg/μl of BSA was loaded in the same nitrocellulose membranes and immunoblotted with C3 antibody. For the chemiluminescence reaction, ECL Femto (Pierce, Thermo Fisher Scientific Inc., Rockford, IL) or Immobilon (Millipore, Schwalbach, Germany) were used.

### Vimentin overlay assay

4 μg of recombinant C3 protein were separated by native 15% PAGE and transferred to nitrocellulose membranes. After Western blot the transfer efficiency was checked with Ponceau S staining. After incubation with blocking buffer (5% powdered milk, in TBST), membranes were probed with 10 μg/ml of purified His-tagged vimentin in blocking buffer for 1 h at 4 °C. After extensive washing with TBST, membranes were incubated with C3 antibody or α-His, followed by HRP-conjugated secondary antibody, and detected by ECL. For the chemiluminescence reaction, ECL Femto (Pierce, Thermo Fisher Scientific Inc.) or Immobilon (Millipore, Schwalbach, Germany) were used. All signals were analyzed densitometrically using the KODAK 1D software (KODAK GmbH, Stuttgart, Germany).

### Expression and purification of recombinant mouse vimentin proteins

Plasmids of mouse vimentins provided by Prof. Dr. Yi-Ling Li, Institute of Biomedical Sciences, Genomics Research Center, Academia Sinica, Taipei, Taiwan, were used (Liang *et al.* 55). The plasmids were transformed into BL21(DE3) cells. Induction was with 1 mm isopropyl 1-thio-β-d-galactopyranoside at 37 °C for 3 h. Recombinant vimentin proteins were purified as described by Machery-Nagel for His tag protein purification (Ni-IDA 2000 Packed Columns Protino, Machery-Nagel GmbH and Co. KG, Düren, Germany). Eluted proteins were analyzed by 15% SDS-PAGE, stained with Coomassie Blue, and submitted to Western blot analysis against α-penta-His (catalog number 34660, Qiagen, Hilden, Germany).

### Reproducibility of the experiments and statistics

All experiments were performed independently at least three times. Results from representative experiments are shown in the figures. Graphs and statistical significance were calculated with GraphPad Prism software (version 6, GraphPad Software, Inc., San Diego, CA). When significant differences were found using ANOVA, Dunnett's multiple *t* test was used to compare the control (C3-treated cells) with the other groups (C3-G89I-treated cells, C3-D90G-treated cells, and C3-D90N-treated cells). The statistical significance of differences between treated vimentin-knock-out and wild-type neurons were calculated by the use of a two-sided unpaired Student's *t* test. Values (*n* ≥ 3) are mean ± S.D. Differences were considered to be statistically significant at *p* ≤ 0.05 (* = *p* ≤ 0.05, ** = *p* ≤ 0.01, and *** = *p* ≤ 0.001).

## Author contributions

I. J., A. R., M. H., and G. A. H. conceived and designed the experiments; S. H., A. A., E. O., and A. R. performed the experiments; A. R. and M. H. analyzed the data; I. J., A. R., M. H., and G. A. H. contributed reagents, materials, and analysis tools; A. R., I. J., M. H., and G. A. H. wrote the paper.

## Supplementary Material

Supplemental Data

## Footnotes

2 The abbreviations used are:

C3: C3 exoenzyme derived from *Clostridium botulinum*
RGD: Arg–Gly–Asp
SARS: severe acute respiratory syndrome
ANOVA: analysis of variance
aa: amino acid(s).
