# Supplementary material for: The Rho ADP-ribosylating C3 exoenzyme binds cells via an Arg–Gly–Asp motif
Source: J Biol Chem. 2017 Sep 7;292(43):17668–80. doi: 10.1074/jbc.M117.798231 (PMC5663871; doi:10.1074/jbc.M117.798231)
Supplement: Supplemental Data [file supp_292_43_17668__index.html]

The Rho ADP-ribosylating C3 exoenzyme binds cells via an Arg-Gly-Asp motif — The Rho ADP-ribosylating C3 exoenzyme binds cells via an Arg-Gly-Asp motif — The Rho ADP-ribosylating C3 exoenzyme binds cells via an Arg–Gly–Asp motif — Functional role of RGD motif for C3 — Supplemental Data 

# The Rho ADP-ribosylating C3 exoenzyme binds cells via an Arg–Gly–Asp motif

## Supplemental Data

- Supplemental data (.pdf, 728 KB) - This is the supplemental data.
