# Supplementary material for: The Rho ADP-ribosylating C3 exoenzyme binds cells via an Arg–Gly–Asp motif
Source: J Biol Chem. 2017 Sep 7;292(43):17668–80. doi: 10.1074/jbc.M117.798231 (PMC5663871; doi:10.1074/jbc.M117.798231)
Supplement: Supplemental Data [file 10.1074_M117.798231_jbc.M117.798231-1.pdf]

The Rho ADP-ribosylating C3 exoenzyme binds cells via an Arg–Gly–Asp motif

Astrid Rohrbeck[1], Markus Höltje[2], Andrej Adolf[2], Elisabeth Oms[1], Sandra Hagemann[1], Gudrun Ahnert-Hilger[2] and Ingo Just[1]

[1] Institute of Toxicology, Hannover Medical School, Carl-Neuberg-Str. 1, D-30625 Hannover, Germany
[2] Institute of Integrative Neuroanatomy, Charité-Universitätsmedizin Berlin, Germany

Running Title: *Functional Role of RGD motif for C3*

Correspondence: Astrid Rohrbeck, PhD, Institute of Toxicology,
Hannover Medical School, Carl-Neuberg-Str. 1,
D-30625 Hannover, Germany
Tel.: #49 (0) 511 5322807; Fax.: #49 (0) 511 5322879;
E-mail: rohrbeck.astrid@mh-hannover.de

Supplementary Figures

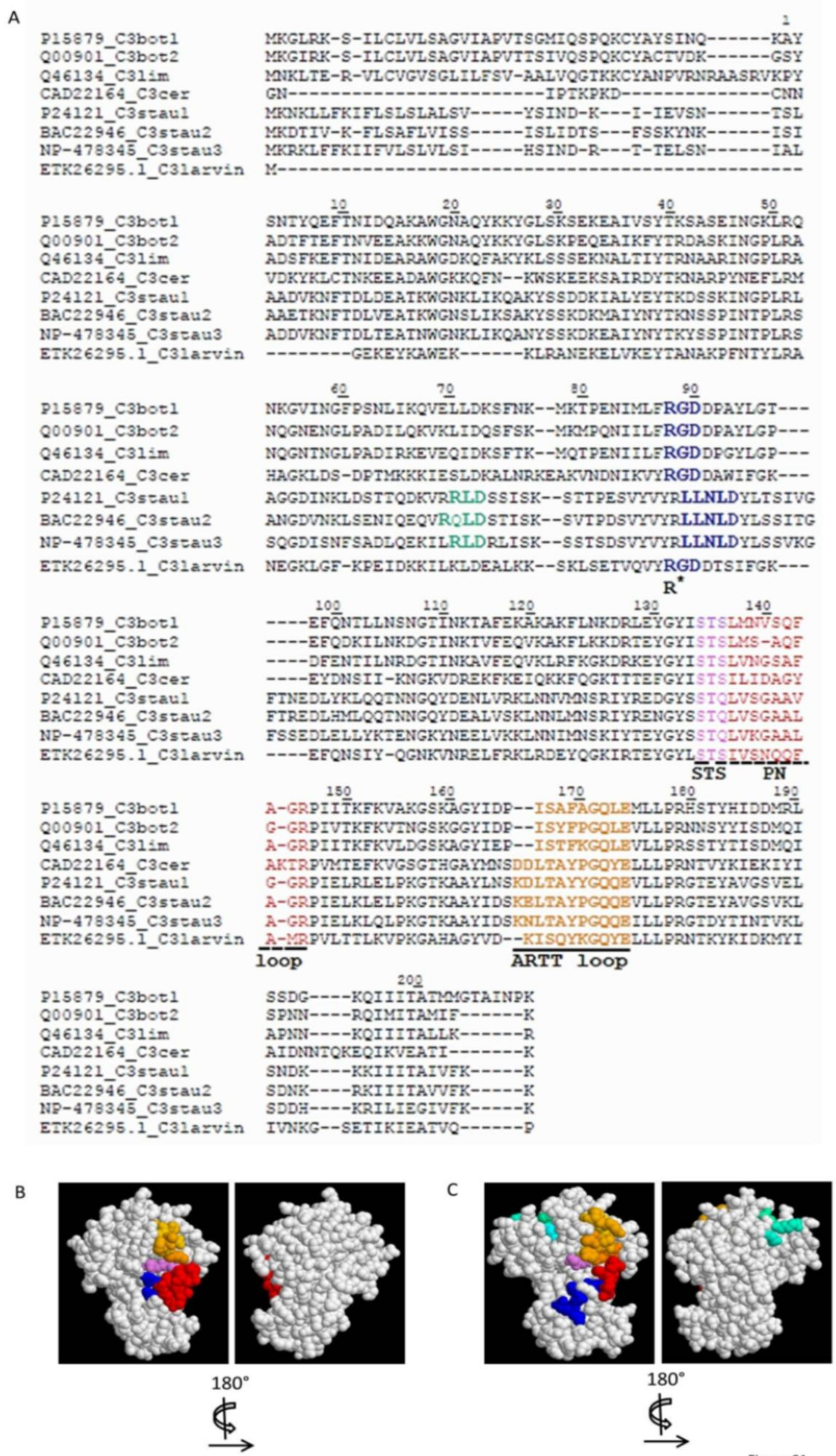

Figure S1

Figure S1: A) ClustalW alignment of C3 exoenzymes. The sequences of the eight C3 ADP-ribosyltransferases are presented with Accession number. Important motifs are highlighted: the RGD/RLD integrin binding motif (blue/green), the NAD-interacting arginine residue (R*), the ADP-ribosylation toxin-turn-turn (ARTT) loop (yellow), the STS-motif (pink) and the phosphate-nicotinamide (PN) loop (red). In C3stau the clathrin box motif is highlighted in bold letter (blue). Molecular coordinates for C3bot1 (B) and C3stau2 (C) were obtained from the Protein Data Bank (http://www.rcsb.org/pdb/) and modeled using Rasmol 2.7.5 (spacefill option). C3bot1 with highlighted important motifs (RGD-motif in blue, ARTT-loop in yellow, STS motif in pink and PN-loop in red) and C3stau2 with RLD-motif in green, LLNLD clathrin box in blue, ARTT-loop in yellow, STS motif in pink and PN-loop in red.

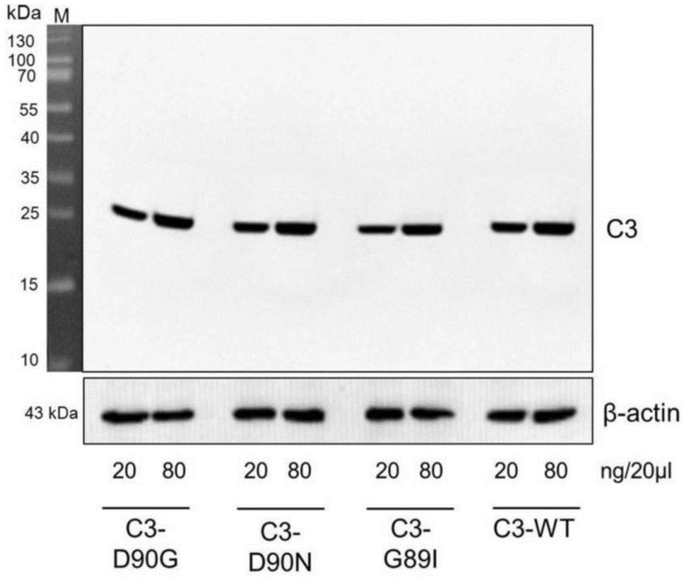

Figure S2

Figure S2: Binding of α-C3bot to C3 and C3-D90G, C3-D90N and C3-G89I. Purified C3 (20 or 80 ng/20 µl) or C3-RGD-mutants (20 or 80 ng/20 µl) were added to HT22 cell lysate and separated through SDS-PAGE and submitted to Western blot analysis against C3 and β-actin.

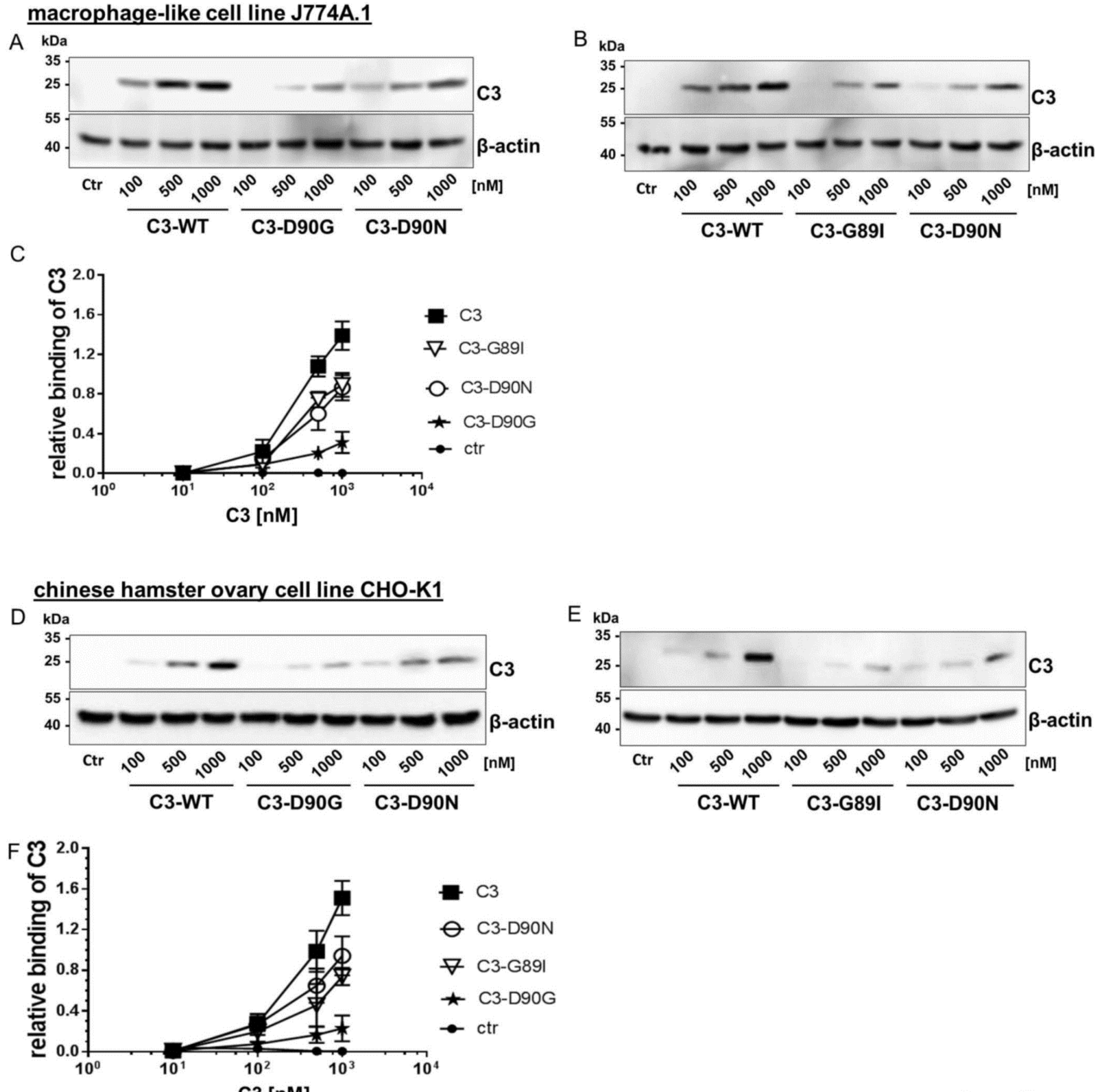

Figure S3

Figure S3: C3-binding assay to intact cells. Cells were exposed to increasing concentrations of C3 and C3-RGD-mutants (C3-D90G and C3-D90N) and C3-G89I (B) for 1 h at 4 °C. Subsequently, cells were stringently washed three times, lysed and submitted to Western blot analysis against C3 and β-actin. (A-C) experiments for the J774A.1 and (D-F) same experiments for the chinese hamster ovary cell line (CHO-K1). For J774A.1 and CHO cell lines results are given as arithmetic means ± SEM from three independent experiments.

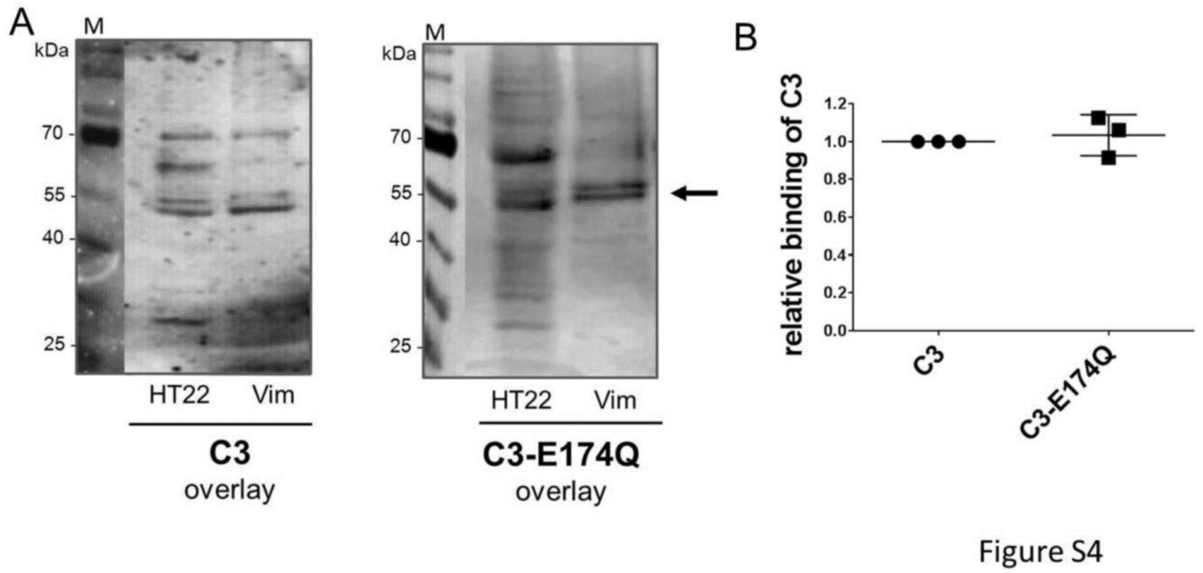

Figure S4:

C3-overlay (binding of C3 to vimentin). A) Binding of C3-E174Q to immobilised vimentin is shown, to rule out unspecific mutational effect on the binding of C3 to vimentin. HT22 cell lysate was used as positive control. B) Densitometrically evaluation of bound C3 is shown (n = 3).
